# Editorial: Dissecting complex gene families to understand their roles in climate-resilience

**DOI:** 10.3389/fpls.2023.1284812

**Published:** 2023-09-12

**Authors:** Venkateswara Rao, Bhanu Prakash Petla, Mehanathan Muthamilarasan

**Affiliations:** ^1^ Department of Plant Sciences, School of Life Sciences, University of Hyderabad, Hyderabad, Telangana, India; ^2^ International Crops Research Institute for the Semi-arid Tropics, Hyderabad, Telangana, India

**Keywords:** genome wide analysis, climate resilience, gene family analysis, stress response, functional characterization

Climate resilience is an important trait that the global plant research community focuses on, for ensuring food and nutritional security. Understanding the genetic determinants underlying this complex trait is key for tweaking the candidate genes, alleles, or QTLs, to enhance climate resilience. Genomics plays a major role in decoding the plant genomes to identify the coding and non-coding elements present within the DNA. Single-copy genes are present scarcely among the coding elements, whereas the remaining genes are encoded as gene families. More than one member of a particular gene family allows the genes to perform a multitude of functions, and their expression is tightly regulated at different levels. Whole genome sequencing reveals the information of genes and their corresponding gene families in the given genome. Characterization of these gene families provides insights into their structural and functional diversity. Thus, recent studies focus on dissecting the structural and functional aspects of gene families, which further identify the candidate genes for downstream characterization. Given the importance of genome-wide analyses in delineating the functional roles of different gene families in imparting climate resilience, the Research Topic invited articles on this aspect of gene family analyses.

Interestingly, the Research Topic received ten original research articles. Suri et al. studied the peptidyl-prolyl cis-trans isomerases (PPIases) in wheat (*Triticum aestivum*) to delineate their role in heat stress response. The authors mainly focused on FKBP (FK506-binding protein), Par (parvulin) and PTPA (phospho-tyrosyl phosphatase activators) gene families that belong to the PPIase superfamily. Through computational approaches, 71 FKBP, 12 Par, and 3 PTPA encoding genes were identified and characterized. Expression analysis of TaFKBP, TaPar and TaPTPA genes in response to heat stress at 50°C showed the putative role of candidate genes in conferring thermotolerance. As wheat is highly sensitive to temperature, Suri et al.have provided leads to understand the PPIase-mediated thermotolerance in this crop. Similarly, Chandra et al. identified two novel transcription factors in wheat, namely, TaZHD1 and TaZHD10, using a comparative genomic approach. These genes belong to the zinc finger homeodomain class of transcription factors and play a major role in leaf rolling during moisture stress conditions. During drought stress, a significant upregulation of TaZHD10 was observed compared to TaZHD1, indicating their involvement in stress-induced leaf rolling. Further, the authors have performed structural and functional characterization of these two genes using *in silico* tools to gain further insights. Altogether, the study provided novel insights into the role of TaZHD1 and TaZHD10 in promoting leaf rolling during moisture stress in wheat, which can further be manipulated for enhanced tolerance to this stress.

Protein kinases play a prominent role in regulating plants’ growth, development, and stress response. Given their importance, studies on identifying and characterizing kinases using whole genome sequencing data are rising. In this context, Santos et al. have identified the kinase-encoding genes of rubber (*Hevea brasiliensis*) to delineate their role in abiotic stress responses. The study identified 2842 protein kinase-encoding genes, which were classified into 20 different groups and 122 families. These genes and their corresponding proteins were characterized using different *in silico* tools to study their chromosomal localization, physicochemical properties, subcellular localization, duplication and divergence patterns, and gene ontology annotation. Further, *in silico* expression profiling was performed using the publicly available RNA-seq data and a co-expression network was constructed. Thus, the study has identified candidate protein kinases that can further be functionally characterized to study their involvement in abiotic stress response. Similarly, Feng et al. studied a class of protein kinases named sucrose non-fermenting-1 (SNF1)-related kinases (SnRKs) in maize (*Zea mays*) for their response to abiotic stresses. A total of 60 SnRK-encoding genes were identified by the authors, which were further studied for their evolutionary relationships, chromosome locations, gene structures, conserved motifs, and *cis*-elements in promoter regions. Also, homology modelling of SnRK proteins was performed, followed by *in silico* expression profiling using the data retrieved from the MaizeGDB database. To validate the findings, the authors treated a maize inbred line (B73) with dehydration and salt stress and the expression pattern of candidate SnRK genes was analyzed. The study, overall, provided insights into the structure and organization of the SnRK gene family in the maize genome and pinpointed candidate genes that can further be studied to understand their role in abiotic stress response.

Metal transporters are reportedly involved in regulating abiotic stresses besides their role in controlling growth and development. Magnesium transporter proteins (MGTs) are on such transporter known for their involvement in response to adverse environmental conditions. In this direction, Tang et al. studied the MGT genes in wheat. Their study identified 24 genes, which were analyzed for phylogenetic relationships, gene and protein structures, chromosomal localization, and organization of *cis-*elements in the promoter region of each gene. In addition, the authors have performed *in silico* expression profiling using publicly available RNA-seq data, and to further validate the findings, qRT-PCR was performed for candidate genes in wheat subjected to different stresses and hormone treatment. Wheat plants subjected to magnesium deficiency, aluminium chloride stress, and abscisic acid treatment were used for qRT-PCR-based expression profiling. The expression pattern was studied in root and shoot tissues of plants subjected to different treatments, and the expression values were compared with that of control (untreated) plants. The authors have observed a differential expression of candidate genes during the stress and hormone treatments compared to control plants, and their study showed the putative involvement of *MGT1B* in maintaining magnesium homeostasis.

Bcl-2-associated athanogene (BAG) proteins regulate growth and development, senescence, and response to environmental stimuli. Given this, Gu et al. studied the structure and organization of BAG proteins and their encoding genes in tobacco (*Nicotiana tabacum*). The study identified 19 BAG proteins in the tobacco genome, which were further characterized for their three-dimensional structures and phylogenetic relationships. Additionally, the authors used qRT-PCR to identify the genes regulating leaf senescence. From this data, *BAG5c* was chosen for further characterization. To confirm the role of BAG5c in regulating senescence, *bag5c* knock-down lines were generated using virus-induced gene silencing approach. The authors have observed a downregulated expression of *NtCP1*, *NtSEN4*, and *NtSAG12* in BAG5c-silenced plants, indicating the positive role of this gene in regulating leaf senescence. Analyzing the subcellular localization revealed the presence of this protein in the cell wall and nucleus. Further, yeast two-hybrid assay indicated the interaction of NtBAG5c and HSP70 in yeast cells. Similarly, in poplar (*Populus trichocarpa*), Liao et al. studied the structure and organization of the laccase gene family. Laccases play roles in lignin biosynthesis, thus gaining importance in biofuel research. The study revealed the presence of 53 laccase-encoding genes in the poplar genome, which are further analyzed using different computational approaches. This includes evolutionary studies, physical mapping, phylogenetic classification, gene structure analysis and motif distribution, domain architecture analysis, and comparative mapping. Also, RNA-seq-based expression profiling was performed to identify the candidate genes, which were further analyzed using qRT-PCR in 84K poplar trees (*P. alba* × *P. glandulosa*). The results suggested the putative involvement of *LAC23* in the lignification process, and to confirm this, the authors generated *proLAC23::LAC23-eGFP* lines. Expression and localization of LAC23 and lignin content showed a strong correlation between each other. The study provided clues on the role of LAC23 in lignin synthesis, thus having a role in realizing the biofuel potential of this important tree species.

Two studies used high-throughput transcriptomics and proteomics tools to identify stress-specific genes and proteins in the marine alga (*Dunaliella salina*) and rice (*Oryza sativa*), respectively. Ramachandran et al. subjected *D. salina* to different salt concentrations to understand the physiological, biochemical, and molecular changes underlying hypersaline tolerance. Similarly, Kumar et al. subjected three cultivars of rice, *viz*., Moroberekan (heat sensitive), IR64 (moderately heat tolerant), and Nagina22 (heat tolerant), to heat stress (short- and long-term), followed by protein isolation from anthers and mass spectrometry (LC-MS/MS). The study identified a repertoire of differentially expressed proteins in anthers during heat stress, which can further be used for imparting durable heat stress tolerance in rice using breeding and transgenic approaches. In another microalga, *Chlamydomonas reinhardtii*, Yadav et al. have used two cyclic electron transport mutants, namely PGRL1 (Proton Gradient Regulation) and PGR5, to understand their role in the assembly and regulation of photosynthetic complexes under high light stress. In addition to biophysical and biochemical analysis of the mutants, the authors performed total proteome analysis using the nLC-MS/MS approach. The data suggested the involvement of cyclic electron transport in maintaining photosynthesis and photoprotection during high light stress. Altogether, the study provides insights into the role of cyclic electron transport in maintaining thylakoid protein abundance and super-complex organization under high light conditions.

In conclusion, the Research Topic has received insightful research contributions showing the role of novel genes in conferring climate resilience. Interestingly, the articles ranged from microalgae to land plants, covering different aspects of dissecting the stress tolerance trait in these organisms. With the advent of next-generation and third-generation sequencing platforms, sequencing complex genomes is now possible, resulting in the release of genome information in public databases. The availability of genome sequencing information enables the researchers to dissect candidate gene families to understand their role in conferring climate resilience. However, besides relying on fundamental bioinformatic analyses and expression profiling, the researchers should focus on the functional characterization of candidate genes to delineate their precise roles during stress incidence and endurance. Also, focus shall be given to studying the involvement of candidate genes in conferring tolerance to multiple (combined or consequential) stresses that impact crop yield and productivity in field conditions. Such information can further be translated to enhance tolerance to individual and multiple stresses in crop plants using advanced approaches such as genomics-assisted breeding, transgene technology, and genome editing.

## Author contributions

VR: Writing – original draft. BP: Writing – original draft. MM: Writing – original draft, Writing – review & editing.

